# Presence of *Acanthamoeba* and diversified bacterial flora in poorly maintained contact lens cases

**DOI:** 10.1038/s41598-020-69554-2

**Published:** 2020-07-28

**Authors:** Dai Miyazaki, Hiroshi Eguchi, Tomomi Kuwahara, Haruyuki Nakayama-Imaohji, Masamaru Inaba, Motozumi Itoi, Kiichi Ueda, Yuichi Ohashi, Kazushige Sado, Satoshi Mizutani, Hitoshi Miyamoto, Shin-ichi Sasaki, Yumiko Shimizu, Yoshitsugu Inoue

**Affiliations:** 1 Division of Ophthalmology and Visual Science, Faculty of Medicine, Tottori University, 36-1 Nishi-cho, Yonago, Tottori 683-8504 Japan; 2 Department of Ophthalmology, Faculty of Medicine, Kindai University, Higashiōsaka, Japan; 3 Department of Microbiology, Faculty of Medicine, Kagawa University, 1750-1 Miki, Kagawa, 761-0793 Japan; 4 Inaba Eye Clinic, Osaka, Japan; 5 Dougenzaka Itoi Eye Clinic, Dogenzaka Shibuya-ku, Tokyo, Japan; 6 Ueda Eye Clinic, Shimonoseki, Yamaguchi Japan; 7 School of Medicine, Faculty of Medicine, Ehime University, Matsuyama, Japan; 8 Sado Eye Clinic, Sendai, Miyagi Japan; 9 Mizutani Eye Clinic, Nagoya, Japan; 10 Department of Clinical Laboratory, Ehime University Hospital, Matsuyama, Japan

**Keywords:** Disease prevention, Clinical microbiology, Epidemiology, Predictive markers, Infection

## Abstract

*Acanthamoeba* can cause visually destructive *Acanthamoeba* keratitis (AK) in contact lens (CL) users. The purpose of this study was to determine whether *Acanthamoeba* was present in the CL cases of CL wearers and to develop techniques to prevent the contaminations. To accomplish this, 512 CL case samples were collected from 305 healthy CL wearers. Using real-time PCR, *Acanthamoeba* DNA was detected in 19.1% of CL cases, however their presence was not directly associated with poor CL case care. Instead, the presence of *Acanthamoeba* DNA was associated with significant levels of many different bacterial species. When the CL cases underwent metagenomic analysis, the most abundant bacterial orders were *Enterobacteriales* followed by *Burkholderiales, Pseudomonadales,* and *Flavobacteriales*. The presence of *Acanthamoeba* was characterized by *Propionibacterium acnes* and *Rothia aeria* and was also associated with an increase in the α diversity. Collectively, *Acanthamoeba* contamination occurs when a diversified bacterial flora is present in CL cases. This can effectively be prevented by careful and thorough CL case care.

## Introduction

*Acanthamoeba* is a free-living amoeba and is a representative pathogen that can cause keratitis. *Acanthamoeba* keratitis (AK) is a visually devastating disease and is refractory to conventional treatments. Thus, AK often requires corneal transplantation. *Acanthamoeba* can also cause granulomatous amoebic encephalitis (GAE). Although the incidence of GAE is rare, the mortality rate is almost 90% because of difficulties in diagnosing and treating it^1^.

AK is known to have strong associations with poor care of contact lenses (CL) and CL cases. When CL users engage in water activities, e.g., bathing or swimming, while wearing CL, there is a significant risk of AK^2,3^.

*Acanthamoeba* is a ubiquitous microorganism that is found in water supply systems, water sources including rivers, lakes, swimming pools, and also in soil^4,5^. It has been reported that *Acanthamoeba* contamination of the rivers supplying water to the population was the cause of increased AK occurrences^6^. For example, the river water supply in Iran was contaminated with *Acanthamoeba*, and almost 22% of river samples were PCR-positive for *Acanthamoeba*^6^. In an analysis of the drinking water storage towers of Paris, approximately 20 to 25 *Acanthamoebae* were detected per liter^1^. Thus, tap water can pose risks for AK contact together with inappropriate CL care for the general population without engagement in water activity^7,8^.

There are many opportunities for *Acanthamoeba* to reach the eyes. However, most CL users do not develop AK even though they are exposed to *Acanthamoeba* on daily basis from the environment.

The purpose of this study was to determine whether poor CL and CL case care leads to contamination. To accomplish this, we cultured CL case samples from 305 CL wearers to determine the population of micro-organisms present in the CL cases, and conducted metagenomic analysis using real-time PCR and next-generation sequencing of DNA. We shall show that the presence of *Acanthamoeba* DNA was not directly associated with poor CL case care, but it was associated with a large number of diversified microbial community which was directly related to poor CL case care.

## Results

We studied 512 CL case samples of 305 CL users who visited five CL clinic for regular examinations throughout Japan from June to October of 2010. Of the 305 CL users, 237 users used disposable soft contact lens and 68 used rigid gas permeable lens. The mean age of the wearers was 37.7 ± 12.7 years. The majority of the users were women (259, 73.8%).

**Figure 1:**
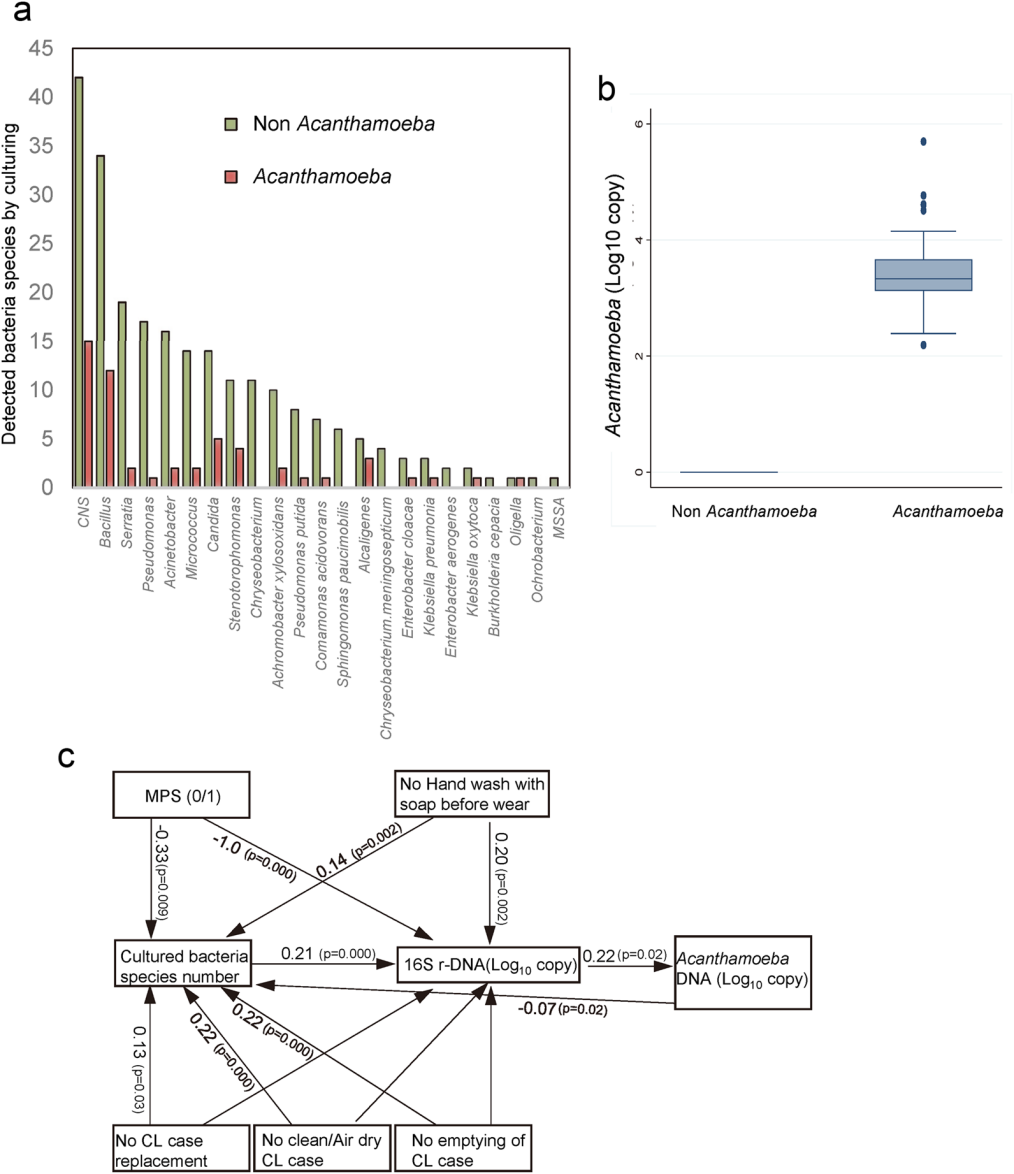
Association of bacterial and *Acanthamoeba* contaminations with poor contact lens (CL) and CL case care. **a** *Acanthamoeba* DNA detection in CL cases (log_10_ copy numbers). **b** Bacterial and fungal contamination assessed by culturing samples from CL cases with or without *Acanthamoeba* contamination. *Acanthamoeba* contamination was assessed by presence or absence of *Acanthamoeba* DNA by real-time PCR. The most prevalent culture-detected species was the coagulase negative *staphylococci* (CNS). **c** Association of microbial and *Acanthamoeba* contamination with poor contact lens case care by covariance structure analyses. Significant associations with bacterial contaminations were observed for “hand wash with soap” (0 to 4), “no regular CL case replacement” (0 to 5), “no clean and air dry of CL case” (0 to 4), and “emptying of CL cases” (0 to 4). Arrows with coefficient (number) indicate significant associations. Fitting indices were RMSEA: 0.000 and CFI: 1.000. For comparison of the effects of CL case care habits, the coding was standardized to a mean of 0 and SD of 1 for covariance structure analyses. Coefficient values and significances were calculated after correction of clusters of each CL user.

We tested for *Acanthamoeba*, bacterial, and fungal contaminations by real-time PCR and culturing the contents of the CL cases (Fig. 1b). *Acanthamoeba* DNA was detected in 19.1% of the cases, and the mean copy number was 3.4 ± 0.5 log_10_ units (Fig. 1a). There were 32.5% of the CL cases that were culture positive for bacteria, and 52.4% of these were gram negative bacteria. The most frequent microbe was coagulase-negative *Staphylococci* followed by *Bacillus sp*, and *Serratia sp.*

### Association of bacterial contamination with poor CL care

We examined whether poor CL case care had caused the contaminations by covariance structure analysis (Fig. 1c). The CL care scores obtained from the questionnaire were standardized (mean 0, SD = 1) for determining the relationships. The results showed significant associations between inappropriate CL case care and the number of bacterial species and copy numbers of 16S r-DNA.

Significant associations were found between the degree of bacterial contamination and the failure of handwashing with soap; there was a 0.2 log_10_ unit increase in the copy numbers of 16S r-DNA (*P* = 0.002) and 0.14 increase in the number of cultured bacterial species (*P* = 0.002). Failure to clean and air dry the CL case was associated with a 0.22 increase in the number of bacterial species (*P* = 0.000). Failure to replace the CL cases with new cases resulted in a 0.22 log_10_ unit increase in the number of bacterial species (*P* = 0.000). Failure to empty the CL cases without drying was positively associated with an increase in the number of bacterial species. These findings indicated that cleaning and air drying the CL cases without wetting will decrease the number and diversity of the bacterial species.

The use of multi-purpose CL solutions was associated 1.0 log_10_ unit reduction in the copy number of 16S r-DNA (*P* = 0.000) and a 0.33 log_10_ unit reduction in the number of cultured bacterial species (*P* = 0.009). These findings indicated the beneficial use of multi-purpose CL solutions to prevent contamination.

In contrast, none of the CL care habits were directly associated with the copy number of the *Acanthamoeba* DNA.

### Association of *Acanthamoeba* presence with bacterial contamination

We then examined whether *Acanthamoeba* presence may be related to bacterial contamination. The level of *Acanthamoeba* was quantified by real-time PCR and assessed for its associations with the level of bacterial contamination by linear regression analysis. The copy numbers (log_10_ units) of *Acanthamoeba* was significantly associated with the copy number of 16S r-DNA (coefficient, 0.13; *P* = 0.009). This is important because the copy numbers of 16S r-DNA is significantly associated with the culture-detected numbers of bacteria (coefficient: 0.49, *P* = 0.000).

The significant associations of cultured bacteria species, 16S r-DNA, and *Acanthamoeba* DNA with each type of CL case care were confirmed using covariance structure analyses, and the results are shown as a path diagram (Fig. 1c). The analyses indicated that the number of cultured bacterial species increased the copy numbers of 16S r-DNA which eventually increased the copy numbers of *Acanthamoeba* DNA.

Collectively, we can conclude that the presence of *Acanthamoeba* is associated with higher bacterial contamination which in turn was associated with poorer CL case care.

### *Acanthamoeba* associated microbial community by metagenomic analysis of CL cases

To examine whether a unique microbial community may be associated with the presence of *Acanthamoeba*, we performed a metagenomic analysis using highly contaminated CL cases. Fifty-nine CL cases of 59 users showed more than 5 log_10_ copy numbers of 16S r-DNA/ml, and they were analyzed for the V3-V4 region of the 16S r-DNA sequence. Of these, 18 cases were positive for *Acanthamoeba* DNA (3.7 ± 0.5 log_10_ copy number/ml).

Paired-end reads of 2,989,602 sequences were generated for the 59 samples. After quality filtering and merging the paired-end reads by Usearch, 1,723,422 merged reads were used for the analyses of the bacterial composition. At the kingdom level, 989 operational taxonomy units (OTUs) were identified. Of these, 98.6% were classified at the phylum level, 97.4% at the class level, 92.1% at the order level, 85.9% at the family level, 52.7% at the genus level, and 31.1% at the species level.

The most abundant order was *Burkholderiales* followed by *Enterobacteriales*, *Pseudomonadales*, and *Flavobacteriales* (Supplementary Fig. 1). We then examined whether CL cases may have different microbial profile depending on the presence of *Acanthamoeba*. (Supplementary Fig. 1C).

**Figure 2:**
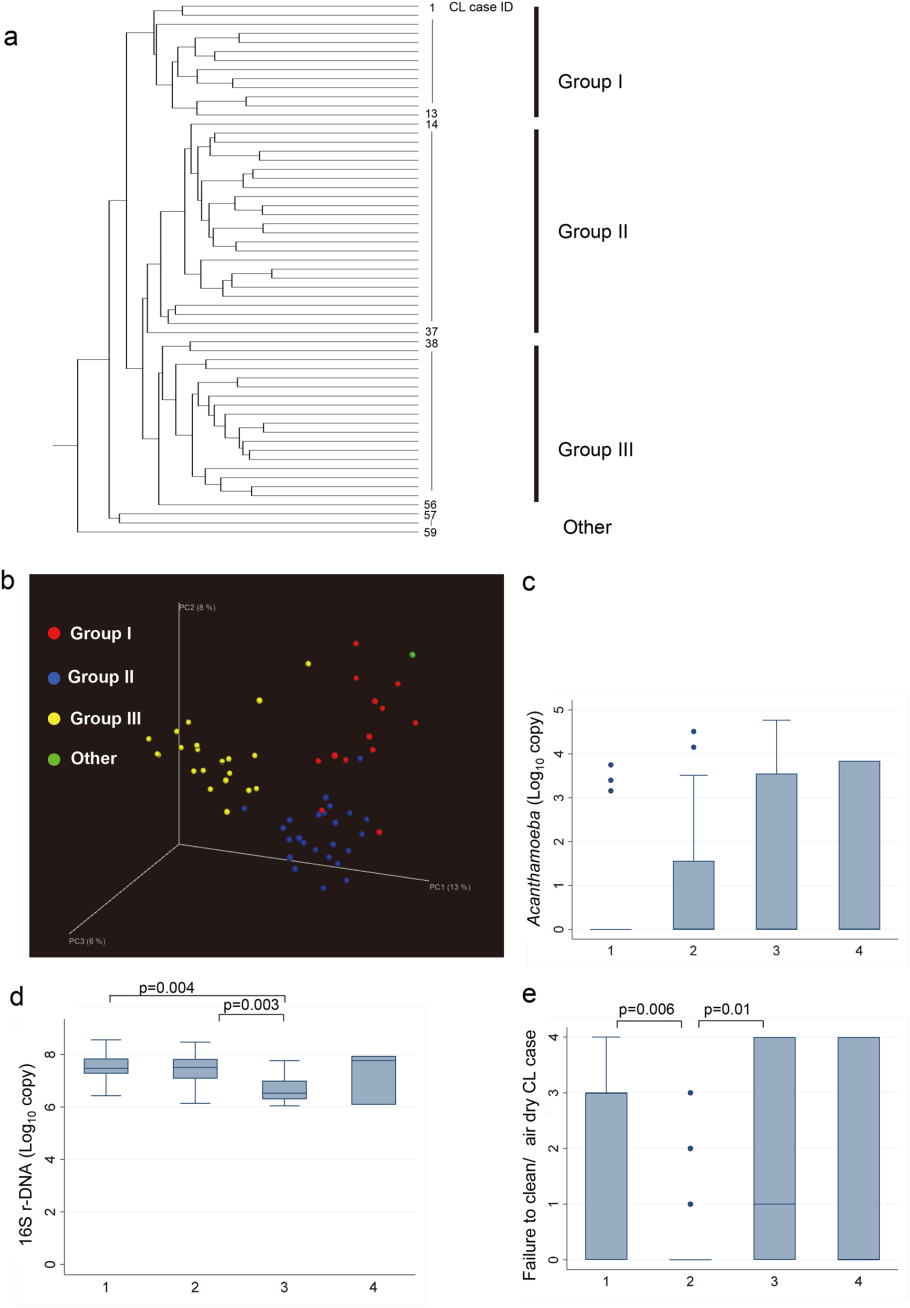
Poor CL case care and copy numbers (in log_10_ units) of 16S r-DNA and *Acanthamoeba* DNA are associated with the CL case microbiome. **a**,**b** Principal coordinate analysis on the relative abundance of bacterial species in CL cases. **c**,**d** Differences of *Acanthamoeba* DNA (**c**) and 16S ribosomal DNA (**d**) depending on groups. ANOVA and Scheffe test. **e** Failure to clean/air dry CL case score is significantly lower for group 2 users. ANOVA and Scheffe test.

To determine the differences of the microbial community in CL cases, OTUs of each CL case were classified by clustering analysis. The unsupervised classification identified four major categories, Categories 1, 2, 3, and others (Fig. 2). We then assessed whether these categories may somehow be associated with the amount of *Acanthamoeba* or bacteria (Fig. 2). *Acanthamoeba* DNA log_10_ units appeared different depending on the category (Jonckheere Terpstra test, *P* = 0.08). The bacterial 16S r-DNA level was significantly different depending on the category, and Category 3 had significantly fewer 16S r-DNA copy numbers.

When poor care CL cases were assessed for associations with the categories, the score for failure to clean and air dry was significantly different depending on the category. Cases in Category 2 had significantly lower score for this failure to clean and air-dry CL cases but with contamination by bacteria.

**Figure 3:**
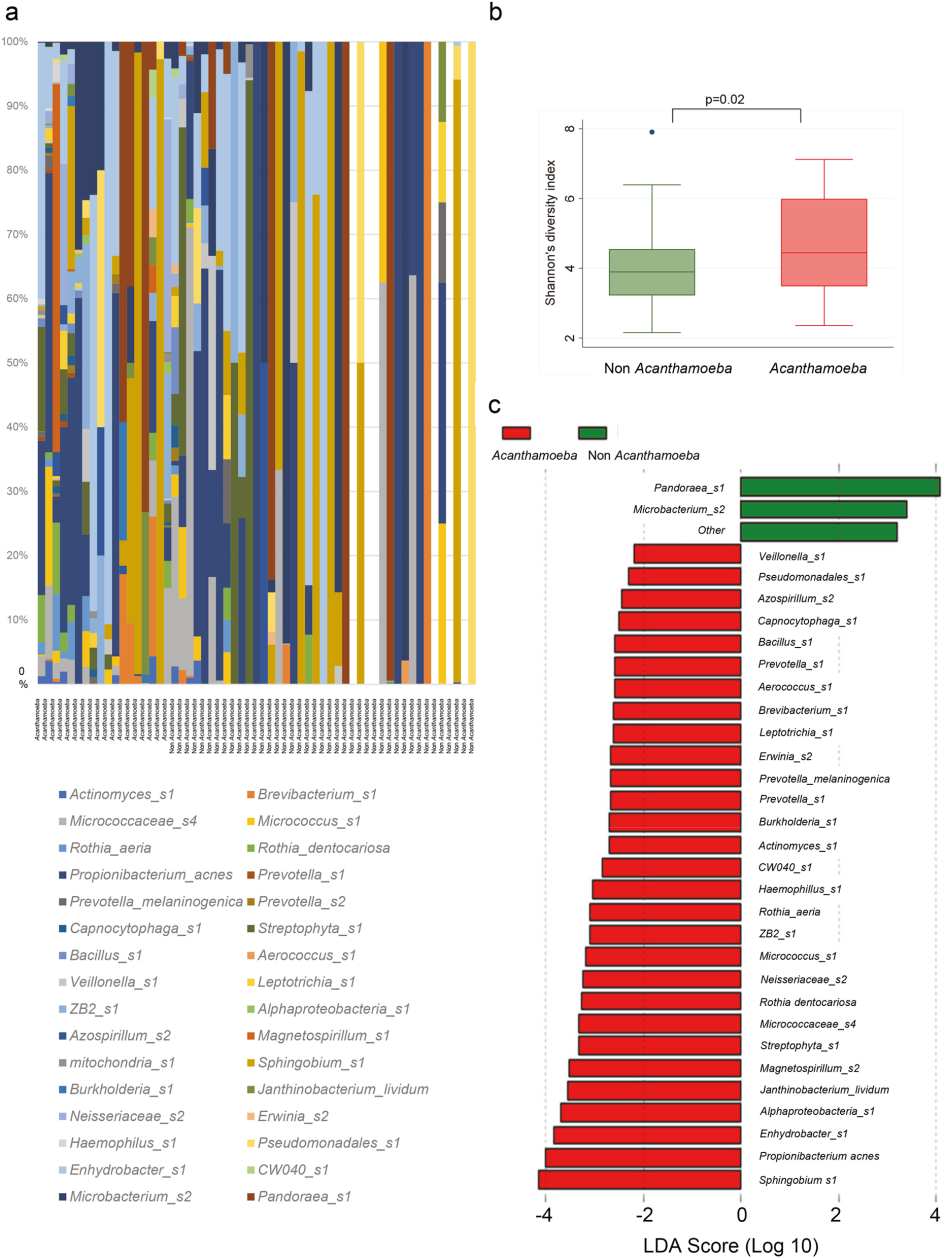
Diversified bacterial species in CL cases with *Acanthamoeba* contamination. **a** Diversity of significantly different bacterial genera in CL cases with or without *Acanthamoeba* contamination. **b** Linear discriminant analysis (LDA) scores of significantly different genera by *Acanthamoeba* contamination by linear discriminant analysis effect size (LefSe) analysis. c. α diversity of CL cases with or without *Acanthamoeba* contamination was calculated as Shannon index. *t*-tests.

We then assessed whether the presence of *Acanthamoeba* may be associated with the microbial diversity in the CL cases (Fig. 3). Shannon’s diversity index was found to be significantly higher for *Acanthamoeba*-present cases (4.9 ± 1.4) compared with non-*Acanthamoeba* cases (4.0 ± 1.2, *P* = 0.01). These findings indicated that the presence of *Acanthamoeba* DNA is associated with an increase in the diversity of the bacterial flora.

To determine whether specific microbial genera were associated with the presence of *Acanthamoeba*, linear discriminant analysis effect size method (LefSe) was conducted (Fig. 3). *Acanthamoeba*-positive cases were significantly associated with a greater diversity of bacterial species. Of these, *Sphingobium* sp., *Propionibacterium acnes*, and *Enhydrobacter* sp. had the highest LDA score which indicated the highly characteristic pathogens in the presence of *Acanthamoeba*. In contrast, non-*Acanthamoeba* cases had only a few genera significantly associated with its presence.

**Figure 4:**
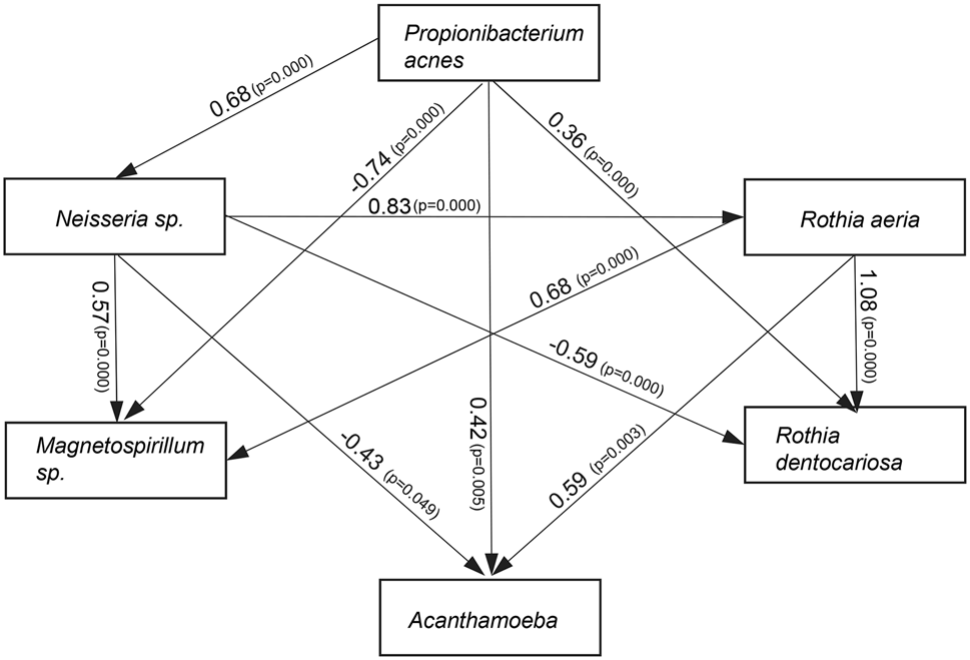
Association of *Acanthamoeba* with core microbial community by covariance structure analyses. Copy number of *Acanthamoeba* (log_10_ units) and microbial community, operational taxonomic units (OTUs) identified by LefSe analysis were assessed for statistical associations. Increased numbers of *Propionibacterium acnes* and *Rothia aeria* significantly higher copy numbers of *Acanthamoeba* in the CL cases. Increased abundance of *Propionibacterium acnes* significantly contributed to the CL case microbial community and was associated positively with *Neisseria sp* and *Rothia dentocariosa*, but negatively with *Magnetospirillum sp*. Standardized co-efficient values are shown by arrows. Positive and negative values indicate positive or negative associations, respectively. Fitting indices were the root mean square error of approximation (RMSEA) 0.000; and CFI, 1.000.

The LefSe analysis indicated the characteristic microbial community that cohabit with *Acanthamoeba*. We further characterized the core community using the microbial species identified by the LefSe analysis. All the bacterial genera reads and *Acanthamoeba* amounts were assessed for direct or indirect relationships using covariance structure analyses (Fig. 4). Of the bacterial genera, significant involvements in *Acanthamoeba*-associated community were observed for *Propionibacterium acnes*, *Neisseria sp, Magnetospirillum sp, Rothia aeria,* and *Rothia dendocariosa*. The abundance of *Propionibacterium acnes* and *Rothia aeria* was significantly increased with an increase in the copy numbers of *Acanthamoeba* in the CL cases.

## Discussion

Hygiene of CL cases is an important factor in preventing CL case-related infectious keratitis including bacterial and *Acanthamoeba* keratitis. However, it remains unclear on which process or habits are specifically important to prevent contaminations of CL cases. The residual fluids in CL cases can support a diverse array of microorganisms including bacteria, *Acanthamoeba*, and fungi.

The 16S r-DNA quantification method and metagenomic analysis showed a diverse microbial community present in the CL cases. Our results showed that poor CL case care can lead to *Acanthamoeba* contamination in 19.1% of the CL cases.

Our findings indicated that the elimination of a diversified bacteria community in the CL cases is the most efficient strategy to prevent *Acanthamoeba* contamination. This is not a direct effect, however, efforts to prevent the development of bacterial community or biofilms should be a feasible and cost-effective means.

To this end, education on CL case care is one simple and effective method. However, information on the advantages and disadvantages of better CL case care is complicated, and many recommendations have been proposed including handwashing, CL case care, choice of multi-purpose solution, and the CL materials. Our data indicated that users should focus on at least three important CL handling steps; air drying of CL cases, hand washing with soap, and periodical CL case replacement.

Based on the calculated standardized co-efficient values, the strength of the associations was similar for the three steps, and they are almost equally recommended. Interestingly, we did not observe any significant effects of rubbing or rinsing of the CL, length of CL wear time, wear period, or CL materials on bacterial or *Acanthamoeba* contamination (data not shown).

There are some possibilities on why the CL materials did not reach statistical significance. First, there were 56 CL products analyzed leading to high heterogeneity of the CL material. However, of the materials of the CLs, we observed the highest positivity of bacterial culture for one rigid gas permeable lens product (Bausch Lomb Ex O_2_, *P* = 0.01, Fisher exact test), and lowest positivity for one soft contact lens (SCL) product (Rohto, i.Q.14, *P* = 0.01). However, both were insignificant after corrections of the multiple comparisons.

Second, our microbiome analysis examined the CL cases and not the CL. This may have reduced the analytical power for parameters relating to CL.

Biofilms provide a significant advantage for microbial residents that cooperatively supply metabolites and nutrients and provide a barrier to resist harsh environmental conditions including chlorine or disinfectants^9^. For example, the highest linear discriminant analysis (LDA) score to discriminate the *Acanthamoeba*-associated community was observed for the *Sphingobium* sp (Fig. 3). This is relevant because *Sphingobium* sp degrade and removes chlorine-based disinfectants^10^. Because the CL case is a limited space environment, not much *Shingobium sp* will be required to change the environment by removing them. This may explain why the amount of *Sphingobium sp* did not have a consistent increase relating to the *Acanthamoeba* amount (Fig. 4).

*Acanthamoeba* feed on bacteria, algae, and yeast which are the other inhabitants in the biofilm^2^. In this setting of a predator system, linear relationships of abundance may well be observed. Collectively, biofilms harboring diverse microorganisms form a niche for *Acanthamoeba* proliferation in an oligotrophic environment.

For culturing the normal conjunctival flora, the coagulase negative *Staphylococcus* (CNS) is the most predominant^11,12^. In our study, the CNS was also most predominant in the cultures of CL cases. When 16S r-DNA sequencing was analyzed for conjunctival flora, *Proteobacteria* was reported as the most abundant phyla (64.4%)^13^. This is also consistent with our metagenomic analysis (Fig. 3). Thus, the microbiome of CL case mirrors the conjunctiva flora. In addition, the normal conjunctival flora appears to be affected by contact lens wear, and skin bacteria are transferred to the ocular surface in CL wearers^11,12^.

What is the source of *Acanthamoeba* contamination in CL case? The drinking water network appears an important source. In the network, *Acanthamoeba* is typically found with the bacterial core community which is predominated by *Pseudomonas* and *Stenotrophomonas*^14,15^. We assume that a harsh environment of disinfectant in CL cases further prompt symbiosis for nutrition, regulation, and protection.

We found a significant number of *Propionibacterium acnes* and *Rothia* sp. in the *Acanthamoeba*-associated community (Fig. 4). *Propionibacterium acnes* was the most abundant and were typically found on the conjunctiva or eyelids^16^. The *Rothia* sp. were also found on the skin or in the mouth, and typically colonize healthy subjects. These associations with gram positive genera were not known previously, and they appear to originate from the body surface of CL users.

There are several limitations to this study. Our analysis of *Acanthamoeba* is based on detection of their DNA and may not represent viable amoeba. Indeed, we could not detect *Acanthamoeba* by culturing using standard CHROMagar Candida lawned with heat-killed *Escherichia coli*^17^. However, the standard short culture does not effectively detect amoeba cysts which entered a viable but non-culturable state^1^. Such a transition can occur in harsh environments such as high chlorine concentrations^18,19^.

For example, chlorinated drinking water with *Acanthamoeba* often become negative by standard culture method^1^. However, *Acanthamoeba* can be detected by extended and more meticulous methods. Taravaud et al. recommended not standard culture, but culturing in 96 well plate after dilution with PYG media for 6 weeks to increase viability^1^. During culture, they used repeated media changing and centrifuging.

We could not apply this time-consuming culturing method. However, we observed very high *Acanthamoeba* DNA amount in CL cases, which far exceeded levels of *Acanthamoeba* concentration in the care solution or ocular surface of normal subjects^20^. This indicates that *Acanthamoeba* appears to have proliferated within and suggests that extended culture might have detected viable *Acanthamoeba*.

In addition, the CL cases were collected from users who visited eye care clinics for regular examinations. Thus, our data may represent those of compliant users and may not reflect the findings of non-compliant users.

Our questionnaire was not previously validated using small samples. This may have reduced the quality of subject-reported data. To address this limitation, we standardized all the coded CL care habits before analysis.

Nevertheless, our data clearly indicated poor CL care is linked to *Acanthamoeba* contamination through the presence of an *Acanthamoeba*-associated core community. Importantly, increased *Acanthamoeba* contamination in CL cases occurs especially when microbial community is diversified and high. We propose that the most effective measure to prevent contamination by *Acanthamoeba* is to reduce the diversification of the flora of CL cases. This can be achieved by three steps; careful CL case drying, periodical replacement of the case, and hand washing before handling the CL. This may reduce risk of *Acanthamoeba* keratitis for CL users.

## Material and methods

### Design and study population

A cross-sectional study of CL users was performed at 5 ophthalmological clinics^17^. The study protocol conformed to the tenets of the Declaration of Helsinki.

A questionnaire was presented to CL users between June to October of 2010^17^. An enrollment invitation was announced and presented to healthy CL users who visited 5 eye clinics, Inaba Eye clinic, Dougenzaka Itoi Eye Clinic, Ueda Eye Clinic, Sado Eye Clinic, and Mizutani Eye Clinic that are located from the north to the southern areas of Japan. The visits were for regular CL maintenance and without ocular surface disease. The request was responded to by 305 CL users after informed consent was obtained. All the participants completed the questionnaire, and the collected information were the sex, age, CL care products, CL maker, frequency of replacement of the contact lenses (2 weeks, 1 month, or 3 months), type of CL (rigid gas permeable lens, and continuous wear), regularity of clinic visits, wearing hours/day and hours/week, and CL care habits including hand washing with soap, drying of CL case, emptying of fluid from CL cases, and replacement of CL cases using questionnaire (Supplementary Table 1). The answer options was structured in an ordered manner as graded in Supplementary Table 1. The questionnaire used was not previously validated first in a small sample of the target subject population.

The coded CL care habits were standardized (mean 0 and SD = 1) and analyzed for covariance structure analysis. Variables, including CL name or CL material, which are irrelevant for ordered grading, were treated as indicating variables.

With the completed questionnaire, CL cases of frequent replacement contact lenses (2 weeks, 1 month, or 3 month), or rigid gas permeable lens were collected.

For double-well CL cases, single well with higher contamination was used for metagenomic analysis, while both samples were used for all the other analyses.

The study protocol was approved by Tottori University Ethics Committee.

### CL case sampling and culturing

The liquid contents of the CL cases were used for the cultures, and stored frozen at −20 °C for extraction of DNA. When the CL cases were dry, elution of 1 ml saline by vortex was used for DNA extraction and culturing. The liquid contents were plated onto 5% sheep blood agar/BTB lactose agar and incubated at 35 °C for 48 h. Isolated colonies were identified by standard microbiological examinations.

For the standard culturing of *Acanthamoeba*, the elution was plated on CHROMagar Candida lawned with *Escherichia coli,* and cultured for 10 days at room temperature.

### Quantitative real-time PCR

DNA was extracted using a QIAamp DNA mini kit (Qiagen, Hilden, Germany), and stored frozen at −80 °C for further analysis by PCR and 16S r-DNA sequencing^17^. *Acanthamoeba* 18S rDNA was amplified using the following primers and probe which distinguished it from other amoebas including *Hartmanella*, *Naegleria*, *Balamuthia*, *Nuclearia*, and *Vahlkampfia*^21,22^.

- Forward: 5′-CGACCAGCGATTAGGAGACG-3′,
- Reverse: 5′-CCGACGCCAAGGACGAC-3′, and.
- TaqMan Probe: 5′-FAM-TGAATACAAAACACCACCATCGGCGC-BHQ.

The copy numbers of the *Acanthamoeba* DNA and 16S r-DNA was calculated by calibration using standard curves generated by serial dilution of cloned DNA fragments of *Acanthamoeba* and 16S r-DNA. The DNA copy numbers were converted to logarithmic (log_10_) units for the statistical analyses.

### 16S r-DNA sequencing and data analyses

The CL case with higher levels of contamination was chosen within each individual, and samples containing more than 10^5^ copies/ml of 16S r-DNA were analyzed for sequencing analysis. The extracted DNA were amplified using KAPA HiFi HS Ready Mix (Nippon Genetics, Tokyo, Japan) and Illumina tagged primers^23^. After the initial amplification, a second PCR was performed to attach Illumina adaptors as well as barcodes that allows for multiplexing. Amplification were performed in 25 μl reactions containing 2.5 μl of diluted template, 12.5 μl of 2 × KAPA HiFi HotStart Ready Mix, and 5 μl each of primers. Thermal cycling consisted of an initial denaturation step (3 min at 95 °C), followed by 25 cycles of denaturation (30 s at 95 °C), annealing (30 s at 55 °C) and 30 s extension at 72 °C. Final extension consisted of 5 min at 72 °C. Amplicons were purified using AMPure XP beads (Beckman Coulter). Sequencing was performed on the Illumina MiSeq platform (MiSeq Reagent Kit ver.3, 600 cycles) according to the manufacturer’s specifications to generate paired-end reads of 300 bases in length in each direction.

Primer sequences were trimmed, and the paired-end reads were merged using fastq-join^24^ with default parameters and processed with QIIME 1.8.0 pipeline^25^. After chimera check by Usearch, 20,000 Illumina reads per sample (average quality score above 20) were randomly selected for further analysis. Using the UCLUST^26^ algorithm built into the QIIME pipeline, sequences were clustered at 97% identity against the Greengenes reference database.

Rarefaction analysis was conducted to evaluate the sampling depth of the sequencing. The microbial α diversity was evaluated by calculating the Shannon index by Qiime2.

Principal coordinate analysis was performed by QIIME and graphically presented by the EMPEROR software (https://biocore.github.io/emperor/)^27^. To determine the differences in the OTU characterization, *Acanthamoeba* DNA contamination, linear discriminant analysis effect size (LefSe) was used.

### Statistical analyses

Data are presented as the means ± standard deviations. Statistical analyses were conducted with the Stata 15 software (Stata Corp, College Station, TX). Mann–Whitney U tests, and ANOVA with post hoc analysis tests were used for multiple comparisons.

Covariance structure analysis was conducted to examine direct and indirect association of inappropriate CL care and bacterial or *Acanthamoeba* contamination. For modelling of CL care habits and contaminations, CL care related-variables were examined for associations with contamination-relating variables using linear regression analysis. Significant variables were chosen for covariance structure analysis. Using these variables, all the possible association (arrows in the path diagram) was tested for goodness of fit.

In the covariance structure analysis (path diagram), numerical variables of two samples from double-well CL case of the same individuals, including bacterial culture, 16S r-DNA copy number, and *Acanthamoeba* copy numbers, were analyzed by treating as nested within the same individuals using generalized covariance structure analysis.

The goodness of fit was evaluated using the root mean squared error of approximation (RMSEA), comparative fit index (CFI), and the Akaike’s information criteria (AIC). A *P* of < 0.05 was considered statistically significant.

## Supplementary information

Supplementary Legends.

Supplementary Figure1.

Supplementary Table1.

## Data Availability

The datasets analyzed during the current study are available from the corresponding author on reasonable request.
