# Supplementary material for: Presence of Acanthamoeba and diversified bacterial flora in poorly maintained contact lens cases
Source: Sci Rep. 2020 Jul 28;10:12595. doi: 10.1038/s41598-020-69554-2 (PMC7387515; doi:10.1038/s41598-020-69554-2)
Supplement: Supplementary file 1 — Supplementary Legends. [file 41598_2020_69554_MOESM1_ESM.docx]

**Supplementary Figure 1.** Presence of bacterial families and genera in the microbiome of CL cases. CL cases contaminated with ≥10^5^ copy numbers/ml of 16S r-DNA underwent metagenomic evaluations.

a: Rarefaction curves of the microbiome of CL cases.

b: Cladograms of the microbiome of CL cases.

c: Abundance of bacterial families and genera in CL cases. *Acanthamoeba* and non-*Acanthamoeba* indicated CL cases which were positive or negative for *Acanthamoeba* DNA, respectively.
