# Supplementary figures and images for: Presence of Acanthamoeba and diversified bacterial flora in poorly maintained contact lens cases
Source: Sci Rep. 2020 Jul 28;10:12595. doi: 10.1038/s41598-020-69554-2 (PMC7387515; doi:10.1038/s41598-020-69554-2)

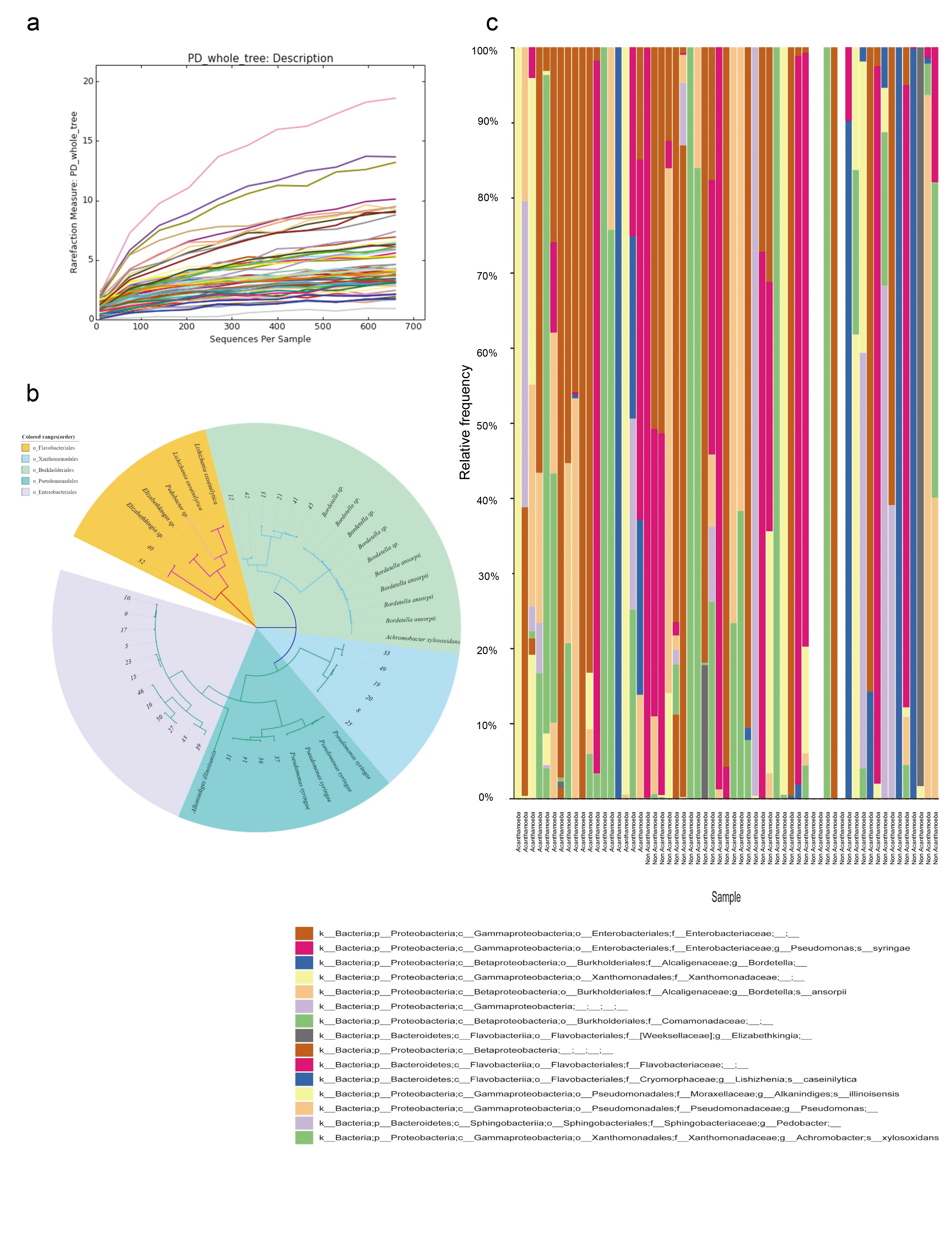

Supplement: Supplementary file 2 — Supplementary Figure1. [file 41598_2020_69554_MOESM2_ESM.tif]
