# Supplementary material for: Presence of Acanthamoeba and diversified bacterial flora in poorly maintained contact lens cases
Source: Sci Rep. 2020 Jul 28;10:12595. doi: 10.1038/s41598-020-69554-2 (PMC7387515; doi:10.1038/s41598-020-69554-2)
Supplement: Supplementary file 3 — Supplementary Table1. [file 41598_2020_69554_MOESM3_ESM.docx]

**Presence of *Acanthamoeba* and diversified bacterial**

**flora in poorly maintained contact lens cases**

Dai Miyazaki^1^, Hiroshi Eguchi^2^, Tomomi Kuwahara^3^, Haruyuki Nakayama-Imaohji^3^, Masamaru Inaba^4^, Motozumi Itoi^5^, Kiichi Ueda^6^, Yuichi Ohashi^7^, Kazushige Sado^8^, Satoshi Mizutani^9^, Hitoshi Miyamoto^10^, Shin-ichi Sasaki^1^, Yumiko Shimizu^1^, Yoshitsugu Inoue^1^

^1^Division of Ophthalmology and Visual Science, Tottori University

^2^Department of Ophthalmology, Kindai University Faculty of Medicine

^3^Department of Microbiology, Faculty of Medicine, Kagawa University, 1750-1 Miki, Kagawa 761-0793, Japan

^4^Inaba Eye Clinic, Osaka Japan

^5^Dougenzaka Itoi Eye Clinic, Dogenzaka Shibuya-ku, Tokyo,

^6^Ueda Eye Clinic, Shimonoseki, Yamaguchi, Japan

^7^School of Medicine Faculty of Medicine, Ehime University

^8^ Sado Eye Clinic, Sendai, Miyagi, Japan

^9^ Mizutani Eye Clinic, Nagoya Japan

^10^ Department of Clinical Laboratory, Ehime University Hospital

**Correspondence to**: Dai Miyazaki, MD, Division of Ophthalmology and Visual Science, Faculty of Medicine, Tottori University, 36-1 Nishi-cho, Yonago Tottori 683-8504, Japan

Tel: 81-859-38-6617

Fax: 81-859-38-6619

E-mail: miyazaki-ttr@umin.ac.jp

**Supplementary Table 1. Questionnaire**

| 1 | Sex | 1. F | 2. M |  |  |  |  |  |
| --- | --- | --- | --- | --- | --- | --- | --- | --- |
| 2 | Age |  |  |  |  |  |  |  |
| 3 | Contact lens type | 1. Soft contact lens | 2. Hard contact lens (Rigid gas permeable lens) |  |  |  |  |  |
| 4 | Name of contact lens and company |  |  |  |  |  |  |  |
| 5 | Contact lens care product name |  |  |  |  |  |  |  |
| 6 | *For soft contact lens users* |  |  |  |  |  |  |  |
|  | Do you use lens care liquid in different contact lens cases from the original? | 1. No | 2. rarely | 3. sometimes | 4. always |  |  |  |
| 7 | *For hard contact lens users* |  |  |  |  |  |  |  |
| 8 | Which solution do you use to store your lens in contact lens case? | 1. Tap water | 2. Homemade solution | 3. Commercial contact lens solution | 4. air dry |  |  |  |
| 9 | Do you wash your hands before contact lens care? | 1. always wash using soap | 2. always wash with water | 3. sometimes wash with soap | 4. sometimes wash with water | 5. No |  |  |
| 10 | Do you clean your contact lens with rubbing? | 1. always rub | 2. mostly rub | 3. sometimes rub | 4. rarely rub | 5. No rubbing |  |  |
| 11 | How do you rub your contact lens? | 1. rub both sides on the palm | 2. rub one side only | 3. rub both sides using fingers |  |  |  |  |
| 12 | Do you rinse your lens before storing in the case? | 1. always rinse | 2. mostly rinse | 3. sometimes rinse | 4. rarely rinse | 5. No |  |  |
| 13 | Do you rinse your lens before wear? | 1. always rinse | 2. mostly rinse | 3. sometimes rinse | 4. rarely rinse | 5. No |  |  |
| 14 | Do you empty your contact lens case after wear? | 1. always empty | 2. mostly empty | 3. sometimes empty | 4. rarely empty | 5. No |  |  |
| 15 | Do you rinse your contact lens case after wear using solution or tap water? | 1. always rinse | 2. mostly rinse | 3. sometimes rinse | 4. rarely rinse | 5. No |  |  |
| 16 | Do you dry your contact lens case without capping after wear? | 1. always dry | 2. mostly dry | 3. sometimes dry | 4. rarely dry | 5. No |  |  |
| 17 | Do you replace your contact lens case regularly? | 1. replace within 1 month | 2. replace within 3 months | 3. replace within 6 months | 4. replace within 12 months | 5. rarely replace | 6. No |  |
| 18 | Duration of use of contact lens case | 1. within 1 month | 2. within 3 months | 3. within 6 months | 4. within 12 months | 5. within 3 years | 6. more than 3 years |  |
| 19 | How many days do you wear your contact lens per week? | 1. every day | 2. 6 days per week | 3. 5 days per week | 4. 3 to 4 days per week | 5. 1 to 2 days per week | 6. irregular | 7. others |
| 20 | How many hours do you wear your contact lens per day? | 1. more than 16 hours | 2. 8 to 16 hours | 3. 4 to 8 hours | 4. less than 4 hours | 5. irregular | 6. others |  |
| 21 | Do you extend wear of your contact lens and sleep in? | 1. yes | 2. No |  |  |  |  |  |
| 22 | How often do you extend lens wear? | 1. a few times per year | 2. a few times per month | 3. a few times per week | 4. almost every day |  |  |  |
| 23 | Do you follow the recommended replacement period of your soft contact lens? | 1. replace within 2 weeks | 2. replace within 3 weeks | 3. replace within 1 month | 4. use more than 1 month |  |  |  |
